# Regio- and diastereoselective synthesis of spiropyrroloquinoxaline grafted indole heterocyclic hybrids and evaluation of their anti-*Mycobacterium tuberculosis* activity†

**DOI:** 10.1039/d0ra02525a

**Published:** 2020-06-19

**Authors:** Natarajan Arumugam, Abdulrahman I. Almansour, Raju Suresh Kumar, Abdul Jaleel Mohammad Ali Al-Aizari, Shatha Ibrahim Alaqeel, Sevgi Kansız, Vagolu Siva Krishna, Dharmarajan Sriram, Necmi Dege

**Affiliations:** Department of Chemistry, College of Science, King Saud University P.O Box 2455 Riyadh 11451 Saudi Arabia anatarajan@ksu.edu.sa aruorgchem@gmail.com +966 4675992 +9664675907; Department of Chemistry, College of Science, King Saud University (034) Riyadh 11495 Saudi Arabia; Department of Fundamental Sciences, Faculty of Engineering, Samsun University Samsun 55420 Turkey; Medicinal Chemistry and Antimycobacterial Research Laboratory, Pharmacy Group, Birla Institute of Technology & Science-Pilani, Hyderabad Campus Jawahar Nagar Hyderabad 500078 Telangana India; Department of Physics, Faculty of Arts and Sciences, Ondokuz Mayıs University Samsun 55139 Turkey

## Abstract

An efficient and eco compatible approach for the regio- and stereoselective synthesis of structurally diverse novel hybrid heterocycles comprising spiropyrrolidine, indenoquinoxaline and indole structural units in excellent yields, has been achieved through a one-pot multicomponent process involving 1,3-dipolar cycloaddition as a key step. The 1,3-dipolar component is the azomethine ylide generated *in situ* from indenoquinoxaline and l-tryptophan and reacts with various substituted β-nitrostyrenes affording the spiroheterocyclic hybrids. The ring system thus created possesses two C–C and three C–N bonds and four adjacent stereogenic carbons, one of which is quaternary and the reaction proceeded with full diastereomeric control. All the synthesized compounds were assayed for their *in vitro* activity against *Mycobacterium tuberculosis* H37Rv using MABA assay. Interestingly, the compound bearing a 2-fluoro substituent on the aryl ring displayed an equipotent activity (MIC 1.56 μg mL^−1^) to ethambutol against *Mycobacterium tuberculosis* H37Rv.

## Abstract 2

An efficient and eco compatible approach for the regio- and stereoselective synthesis of structurally diverse novel spiropyrrolidine tethered indole hybrids in excellent yields employing a one-pot multicomponent 1,3-dipolar cycloaddition strategy.

## Graphical abstract

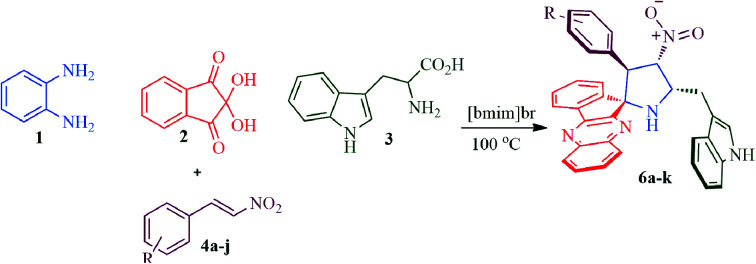

## 1. Introduction

Tuberculosis (TB) is a chronic infectious disease caused by *Mycobacterium tuberculosis* bacteria (MTB), that has become an important world-wide public health problem.^1^ According to the World Health Organization (WHO), approximately 10 million people are believed to be infected with TB annually with almost 1.5 million deaths.^2^ Further, HIV-infected patients have an elevated risk of primary or reactivated tuberculosis, and such an active infectious process may enhance HIV replication and increase the risk of death.^3^ It is estimated that about one third of the world's population is infected with latent tuberculosis. Currently available first line anti-TB medications *viz*., ethambutol, isoniazid, pyrazinamide and rifampicin suffer from associated side-effects, poor efficacy in eradicating dormant pathogens and prolonged treatment.^4^ The second line anti-TB drugs such as bedaquiline, delamanid and pretomanid are generally more toxic, less effective and more expensive than the first line anti-TB drugs. Besides, the existence of multidrug and extensively drug resistant tuberculosis (MDR-TB and XDR-TB) further intensifies the problems connected with TB treatment. As patients could become virtually untreatable with the currently available anti-TB drugs, it is imperative to develop structurally diverse, novel, effective, fast acting and affordable anti-TB drugs having a unique mechanism of action with reduced toxicity profiles, capable of overcoming the resistances posed by MDR-TB and XDR-TB to efficiently combat this disease.

In this perspective, spiro compounds are very attractive structural motif for drug discovery since they are intrinsically three dimensional structures that can facilitate interactions with three dimensional binding sites more easily than planar aromatic ring system as ligand. A large number of spiro compounds found in natural products evolved to interact more efficiently with binding pockets in proteins and have better solubility, a crucial property in the process of drug development. Among them, pyrrolidines embedded in a spiro core are prevalent in several alkaloids and synthetic analogs including spirotryprostatins A and B,^5^ horsfiline,^6^ elacomine,^7^ formosanine,^8^ rhynchophylline,^9^ MI-219, MI-773 and MI-888. These spiropyrrolidine heterocyclic hybrids displayed interesting biological properties including anticancer,^10–12^ antimycobacterial,^13^ anti-inflammatory, anti-microbial,^14^ analgesic,^15^ local anesthetic^16^ and AChE inhibition activities.^17–19^ Earlier studies showed that spiropyrrolidine heterocyclic hybrids displayed significant antimycobacterial activities that are comparable or superior to those of some of the currently employed first line TB drugs.^20^

**Scheme 1:**
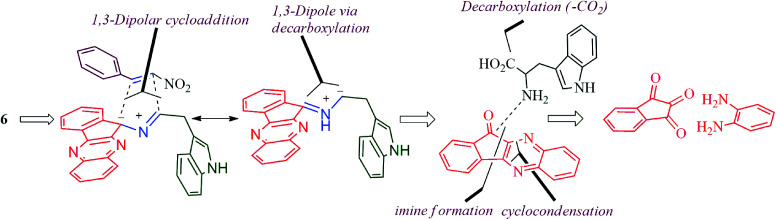
Synthetic strategy for spiropyrrolo-indenoquinoxaline tethered indole analogs.

Despite their biological significances, the syntheses of spiropyrrolidine containing indole side chains have received little attention. In the context of our research in the field of 1,3-dipolar cycloadditions,^21–29^ herein we report an easy access to the target spiropyrrolo-indenoquinoxaline-indole heterocyclic hybrids *via* a one-pot, green synthetic protocol employing a 1,3-dipolar cycloaddition in ionic liquids. The synthetic strategy planned for the preparation of spiropyrrolo-indenoquinoxaline tethered indole heterocyclic hybrid has been described in Scheme 1. The 1,3-dipole derived from l-tryptophan is relatively less explored in the literature and, to best of our knowledge, this is the first report of the synthesis of 4-nitro-3-phenylspiro[indeno[1,2-*b*]quinoxaline-pyrrolidin-5-yl)methyl)-indole *via* one of such a novel class of azomethine ylide, derived from indenoquinoxalinone and l-tryptophan together with their biological evaluation.

## 2. Results and discussion

### 2.1 Chemistry

**Scheme 2:**
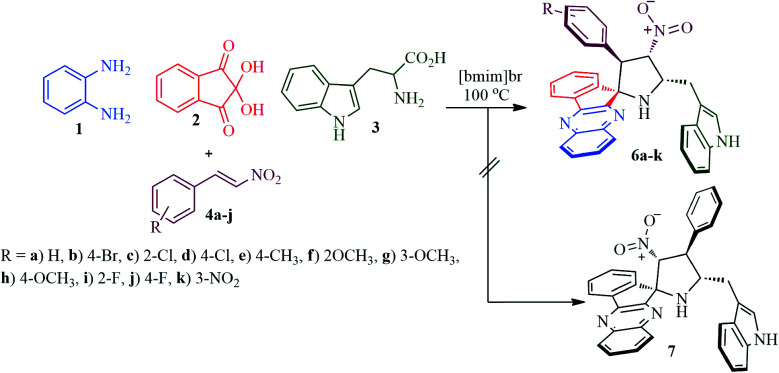
Synthesis of spiropyrrolo-indenoquinoxaline tethered indole heterocyclic hybrids, 6a–k.

Our synthetic study begins with a pilot experiment that involved refluxing a mixture of *o*-pheneylenediamine 1, ninhydrin 2, l-tryptophan 3 and β-nitrostyrene 4k^30^ in methanol for 2 h, which led to the isolation of 3-((4′-nitro-3′-phenylspiro[indeno[1,2-*b*]quinoxaline-11,2′-pyrrolidin]-5′-yl)methyl)-1*H*-indole 6k as a single diastereoisomer in 75% yield. The same reaction was also examined in different solvents including ethanol, acetonitrile, 1,4-dioxane and methanol : 1,4-dioxane (1 : 1 v/v) under reflux. A slightly improved yield of the product 6k (77%) was observed in MeOH : 1,4-dioxane mixture (Table 1, entry 4). Furthermore, the reaction optimization was also investigated in an ionic liquid [bmim]Br, which led to the formation of the desired product in an excellent yield of 86% (Table 1, entry 5) in a short reaction time (Scheme 2). Following the optimization study, all subsequent reactions were effected by heating an equimolar mixture of the reactants in [bmim]Br in an oil bath at 100 °C for 45 min and the products 6 were furnished in excellent yields, whilst the ionic liquid could be recovered and reused by simple drying under vacuum. The other possible regioisomer of 6, *i.e.*, compound 7 was not observed even in traces (Scheme 1). It is pertinent to note that the reaction proceeded in a highly regio- and stereoselective fashion generating two new C–C, three C–N bonds and four adjacent stereocenters, one of which is a spiro carbon, in a single operation. It is noteworthy to mention that the rate of the reaction was accelerated by [bmim]Br^31^ as it possesses electron deficient hydrogen atom which could form hydrogen bonds with heteroatoms of the starting substrates thereby catalyzing the reactions. This multicomponent reaction progressed in a short reaction time with high yield as evidenced by our earlier reports.^32–34^

**Table 1:** Solvent optimization for the synthesis of spiroheterocyclic hybrid, 6k

Entry	Solvents	Time (h)	Yield (%)
1	Ethanol	2	72
2	Methanol	2	75
3	Acetonitrile	2	69
4	Methanol : 1,4-dioxane	2	77
5	[bmim]Br	45 min	86

**Fig. 1:**
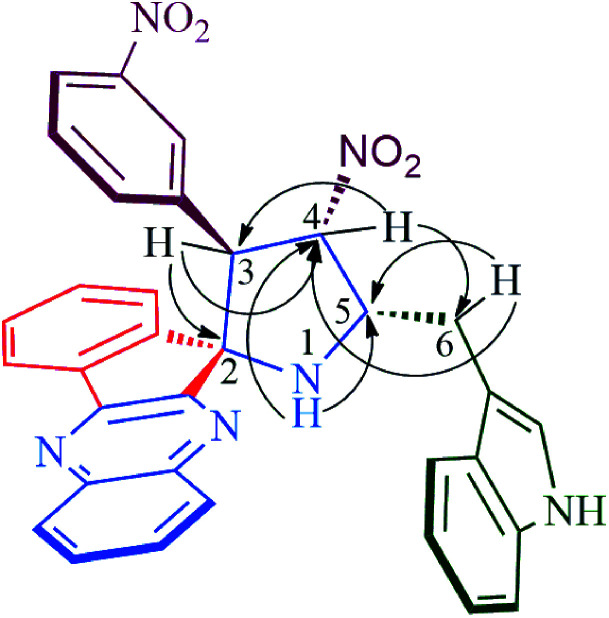
Selected HMBCs of 6k.

**Fig. 2:**
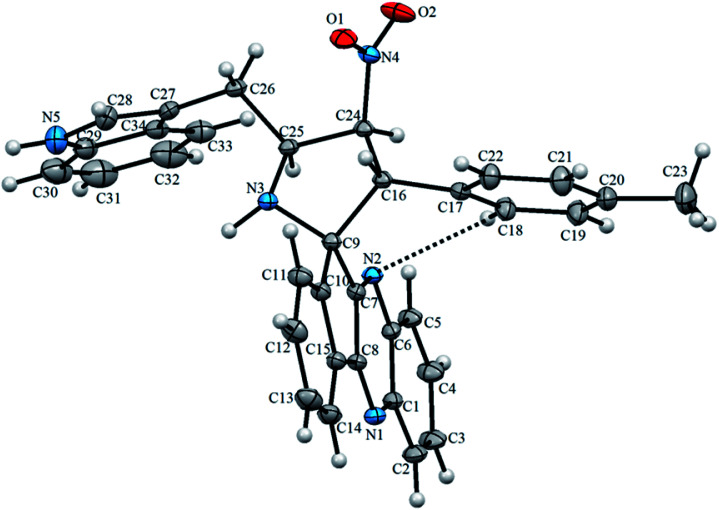
The asymmetric unit of 6e. Dashed line denotes the intramolecular C–H⋯N hydrogen bond.

Structural elucidation of the regioselective spiroheterocyclic hybrids was done carefully using 1D and 2D NMR spectroscopic analysis (*vide* supporting data) as discussed for a representative example, 6k (Fig. S1,†*vide* supporting data). In the ^1^H NMR spectrum, the H-3 hydrogen appear as a doublet at *δ* 5.04 ppm (*J* = 10.0 Hz) which showed H,H-COSY correlation with the triplet at *δ* 6.75 ppm (*J* = 9.0 Hz) being assignable to H-4 hydrogen which in turn shows HMBCs (Fig. 1) with the spirocarbons (C-2) and C-4 at *δ* 72.5 and *δ* 91.0 ppm respectively. The H-4 hydrogen also showed (i) H,H-COSY correlation with a multiplet at *δ* 5.13–5.19 ppm ascribable to H-5 hydrogen(ii) HMBCs with C-3 and C-6 at *δ* 57.3 and *δ* 28.6 ppm respectively. H-5 hydrogen showed H,H-COSY correlation with the doublet of doublets at *δ* 3.00–3.05 and *δ* 3.09–3.13 ppm due to H-6 hydrogens. H-6 showed HMBCs with C-5 and C-4 at *δ* 58.5 and *δ* 91.0 ppm respectively (*vide* supporting data). Finally, the regio- and stereochemistry of the spiro cycloadduct was unambiguously ascertained by single crystal X-ray diffraction analysis of 6e (Fig. 2).^35^

**Fig. 3:**
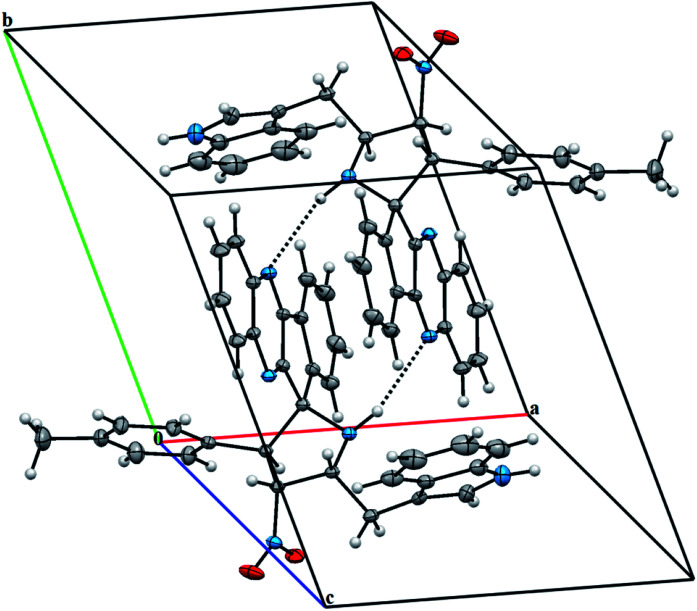
A partial view of the crystal packing of 6e. Dashed lines denote the intermolecular N–H⋯N hydrogen bonds.

The X-ray diffraction data of 6e was collected on STOE IPDS 2 (Stoe Imaging Plate Diffraction System II) diffractometer with graphite-monochromatized Mo-Kα radiation (*λ* = 0.71073 Å).^36^ The crystal structure was solved by SHELXT^37^ and refined anisotropically by using SHELXL17/1 (ref. 38) software. WinGX^39^ software was used to prepare material for publication. An additional solvent molecule (2-butanol) of partial occupancy was removed with the SQUEEZE procedure in PLATON.^40^ The asymmetric unit of 6e compound is shown in Fig. 2 and, crystal data, data collection and structure refinement details are summarized in Table S1† (*vide* supplementary data). Its asymmetric unit contains one independent molecule. The molecular structure is stabilized by the intramolecular C–H⋯N hydrogen bond (Fig. 2). The crystal packing of 6e features C–H⋯O and N–H⋯N hydrogen bonds (Fig. 3 and Table S2,†*vide* supplementary data). The N–O bond lengths [1.208 (2) and 1.198 (3) Å] in the nitro group are close to the values observed for related compounds reported in the literature.^41–43^ The detailed crystallographic and density functional theory (DFT) studies of spiropyrrolidine will be investigated in due course.

**Scheme 3:**
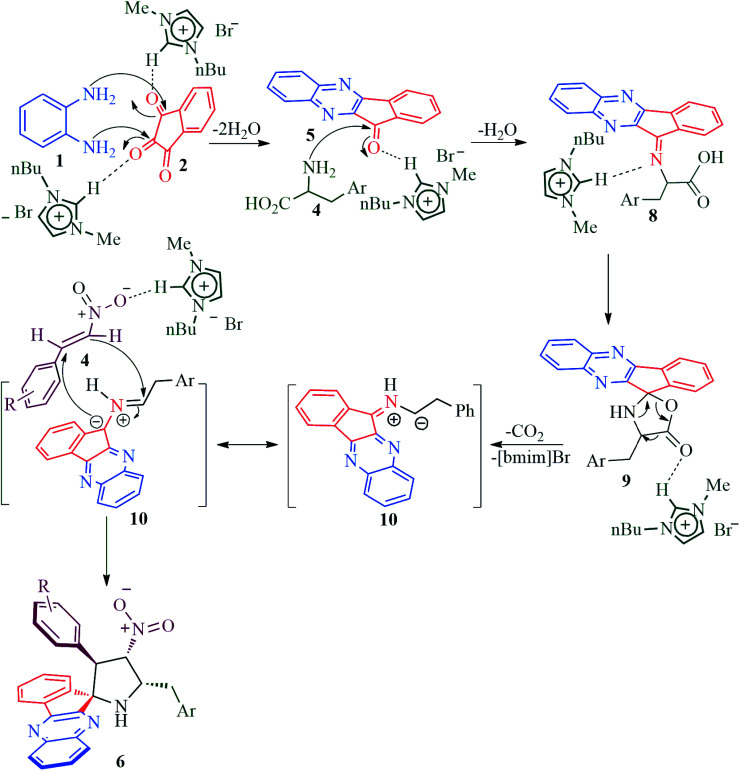
Plausible mechanism for the formation of observed regiochemistry of spiropyrrolidines, 6.

**Scheme 4:**
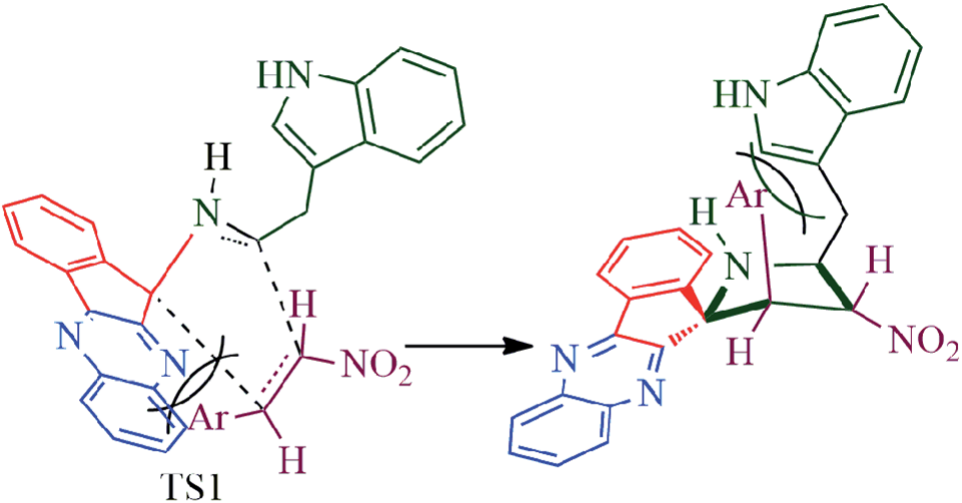
Explanation of unobserved stereoselectivity.

The reaction presumably proceeds through a mechanism proposed in Scheme 3. Thus, the mono ketone 5 initially formed by the reaction of *o*-pheneylenediamine 1 and ninhydrin 2 reacts with l-tryptophan 4 to generate the 1,3-dipole 10*via* imine 8 and isoxazolidinone intermediate 9. Subsequently, the 1,3-dipole component 10 attacks the β-carbon of styrene 4 regioselectively to afford the spirocycloadduct 6 (Scheme 2). Furthermore, the spiroheterocycle 6 was obtained with complete stereoselectivity. The aryl ring substituted to the pyrrolidine ring adjacent to the spiro carbon being *cis* to the quinoxaline unit and *trans* to the nitro group. The transition state (TS1) reveals the unfavorable interaction between the phenyl ring and quinoxaline unit of indenoquinoxaline moiety leading to the unobserved stereoisomer (Scheme 4).

### 2.2 Biology

Compounds 6a–k thus synthesized were evaluated for their *in vitro* antimycobacterial activity against *Mycobacterium tuberculosis* H37Rv by microplate alamar blue assay (MABA) and test results were presented as minimal inhibition concentration (MIC in μg mL^−1^) as described in Table 2. The following three anti-TB leads such as Isoniazid, rifampicin and ethambutol were used as reference standard. Among the synthesized spiropyrrolo-indenoquinoxaline tethered indole heterocyclic hybrids, the four compounds 6b, 6c, 6i and 6j showed moderate to potent activity which is comparable to that of the standard drug, Ethambutol. Compounds 6i with *o*-fluoro and 6j with *p*-fluoro substituent on the aryl ring possesses remarkable activity against MTB with MIC values 1.56 and 3.125 μg mL^−1^, respectively while compounds 6b and 6c bearing *p*-bromo and *o*-chloro substituent on the aryl ring displayed moderate activity against MTB with MIC values 6.25 and 12.5 μg mL^−1^, respectively. Compound 6i bearing 2-fluoro on the aryl ring exhibited equipotent activity as ethambutol (MIC = 1.56 μg mL^−1^). The three potent anti-TB compounds *viz*, 6b, 6i and 6j were tested for their toxicity on normal cell lines. As *Mycobacterium tuberculosis* resides in macrophages, we have tested them on Raw 264.7 macrophage cell lines and these compounds were found to be safe at 50 μg mL^−1^ test concentration. The percentage of inhibition data of these compounds is summarized in Table 2. The above results revealed that spiropyrrolidines possessing halogen substituents on the phenyl ring displayed significant activity, in particular fluoro substituted spiroheterocyclic hybrids displayed excellent activity.

**Table 2:** Yield and MIC values and cytotoxicity data of spirpyrrolidine tethered indenoquinoxaline hybrids 6a–k against *Mycobacterium tuberculosis*

Entry	Compound	Yield (%)	MIC (μg mL^−1^)	Toxicity (% inhibition when tested at 50 μg mL^−1^)
1	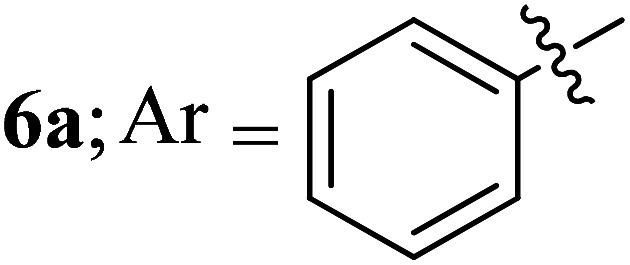	84	>25	
2	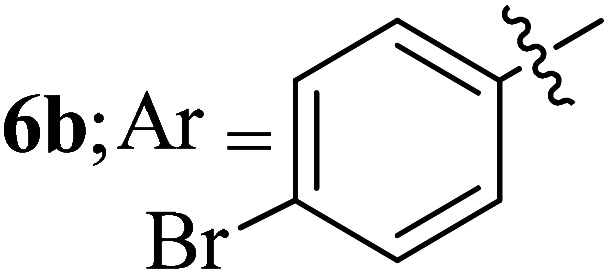	81	6.25	26.15
3	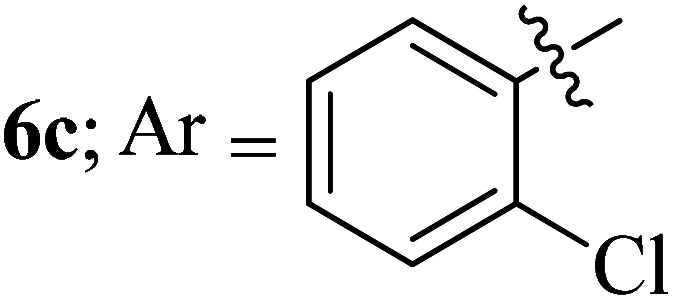	87	12.5	—
4	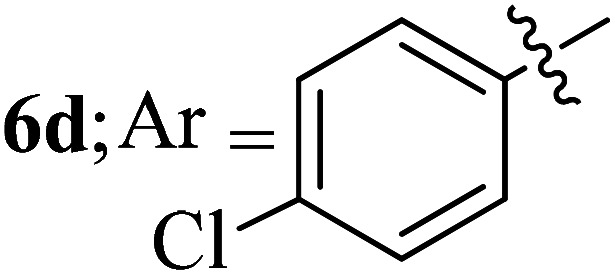	84	25	—
5	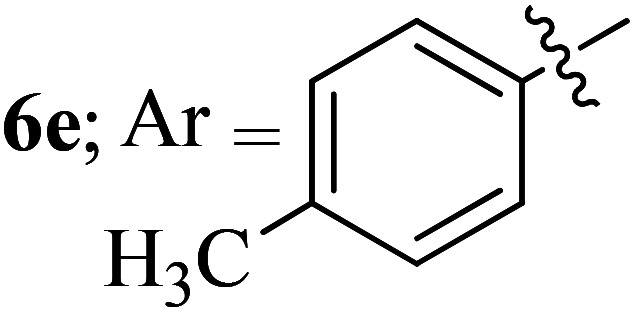	88	>25	—
6	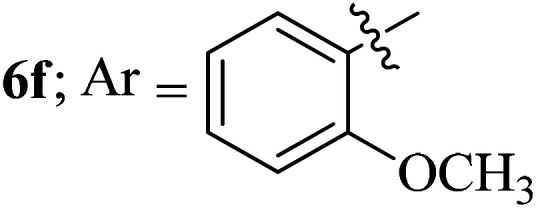	80	>25	—
7	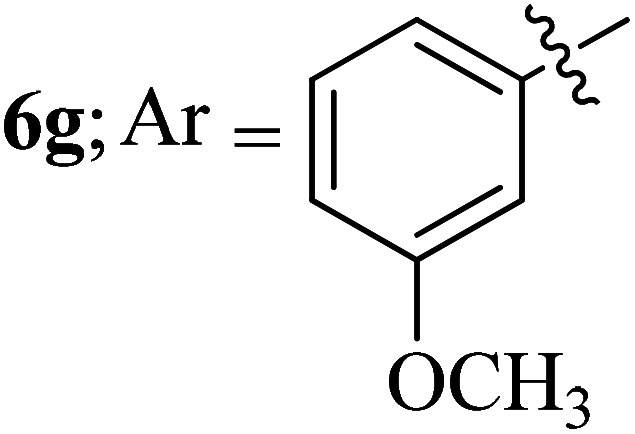	79	>25	—
8	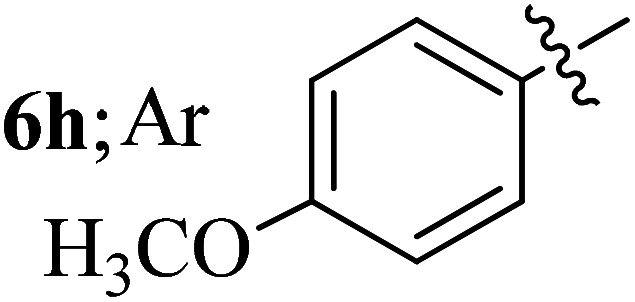	85	>25	—
9	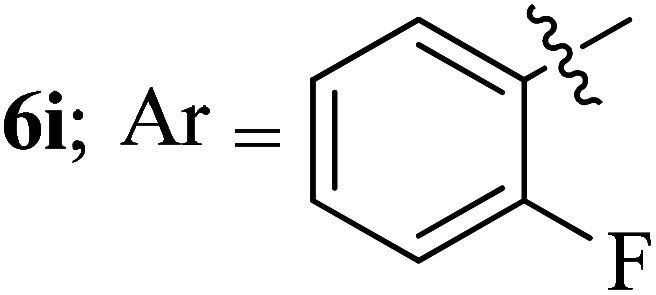	84	1.56	32.40
10	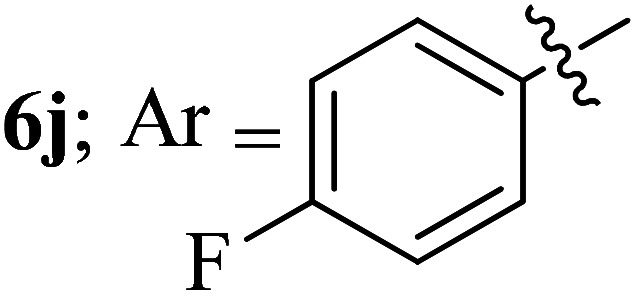	87	3.125	28.02
11	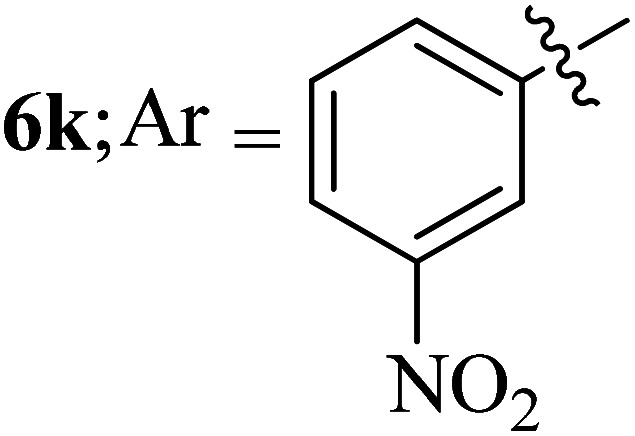	81	>25	—
12	Isoniazid	—	0.05	
13	Rifampicin	—	0.1	
14	Ethambutol	—	1.56	

## 3. Conclusion

The present study describes a regio- and stereoselective synthesis of spiropyrrolo-indenoquinoxaline tethered indole heterocyclic hybrids in excellent yields *via* [bmim]Br acelerated multicomponent cascade reaction protocol. It is pertinent to note that a new class of non-stabilized 1,3-dipole component generated from l-tryptophan and indenoquinoxalinone was employed. The spiroheterocyclic hybrids possess two C–C and three C–N bonds and four contiguous stereocenters, out of which one is a spirocarbon. *In vitro* antimycobacterial activity of these synthesized compounds against *Mycobacterium tuberculosis* H37Rv revealed that the spiroheterocyclic hybrid bearing *o*-fluoro substituent on the phenyl ring (6i) displayed the most potent activity (1.56 μg mL^−1^) and less toxicity on Raw 264.7 macrophage cell lines at 50 μg mL^−1^ concentration suggesting that this compound would be a promising hit for the development of new anti-TB lead compounds.

## 4. Material and methods

### 4.1 Synthesis of spiropyrrolo-indenoquinoxaline tethered indole heterocyclic hybrids, 6a–k

An equimolar mixture of aryldiamine 1, triketone 2, l-tryptophan 3 and β-nitrostyrene 4 in [bmim]Br (200 mg) were heated with stirring at 100 °C for 45 min. After completion of the reaction (TLC), ethyl acetate (10 mL) was added and the reaction mixture was further stirred for 10 min. The ethyl acetate layer was separated and the solvent was removed under reduced pressure to afford 6 in excellent yields.

#### 4.1.1 3-((4′-Nitro-3′-phenylspiro[indeno[1,2-*b*]quinoxaline-11,2′-pyrrolidin]-5′-yl)methyl)-1*H*-indole, 6a

White solid; Mp: 218–220 °C; ^1^H NMR (DMSO-d_6_, 400 MHz): *δ*/ppm 3.05–3.10 (m, 2H), 4.06 (d, *J* = 6.0 Hz, 1H, NH), 4.87 (d, *J* = 10.0 Hz, 1H), 5.09–5.13 (m, 1H), 6.70–6.78 (m, 2H), 6.82–6.91 (m, 3H, ArH), 7.00–7.09 (m, 2H, ArH), 7.25 (d, *J* = 2.4 Hz, 1H, ArH), 7.35 (d, *J* = 8.0 Hz, 1H, ArH), 7.55 (t, *J* = 8.0 Hz, 1H, ArH), 7.70–7.77 (m, 4H, ArH), 7.84 (d, *J* = 8.0 Hz, 1H, ArH), 7.94–7.96 (m, 1H, ArH), 8.16–8.22 (m, 2H, ArH), 10.88 (s, 1H, NH); ^13^C NMR (CDCl_3_, 100 MHz): *δ/*ppm 28.4, 57.3, 57.4, 71.8, 90.5, 110.0, 111.4, 118.3, 120.9, 123.6, 125.2, 127.3, 127.5, 127.7, 127.9, 128.7, 129.2, 129.3, 129.8, 129.9, 132.3, 132.9, 136.2, 136.7, 140.3, 141.4, 147.5, 153.0, 163.5. LC/MS(ESI): *m/z* = 523 (M^+^); anal. calcd for C_33_H_25_N_5_O_2_: C, 75.70; H, 4.81; N, 13.38; found: C, 75.81; H, 4.93; N, 13.49.

#### 4.1.2 3-((3′-(4-Bromophenyl)-4′-nitrospiro[indeno[1,2-*b*]quinoxaline-11,2′-pyrrolidin]-5′-yl)methyl)-1*H*-indole, 6b

White solid; Mp: 245–247 °C; ^1^H NMR (DMSO-d_6_, 400 MHz): *δ*/ppm 3.02–3.12 (m, 2H), 4.07 (d, *J* = 6.0 Hz, 1H, NH), 4.88 (d, *J* = 10.0 Hz, 1H), 5.07–5.10 (m, 1H), 6.68–6.75 (m, 3H, ArH), 7.00–7.12 (m, 4H, ArH), 7.24 (s, 1H, ArH), 7.34 (d, *J* = 8.0 Hz, 1H, ArH), 7.56 (t, *J* = 8.0 Hz, 1H, ArH), 7.69–7.79 (m, 4H, ArH), 7.86 (d, *J* = 8.0 Hz, 1H, ArH), 7.96 (d, *J* = 8.0 Hz, 1H, ArH), 8.16–8.21 (m, 2H, ArH), 10.87 (s, 1H, NH); ^13^C NMR (CDCl_3_, 100 MHz): *δ/*ppm 28.3, 56.6, 57.3, 71.7, 90.3, 109.9, 111.4, 118.3, 118.4, 120.9, 121.4, 123.6, 125.3, 127.3, 128.7, 129.2, 129.3, 129.9, 130.0, 130.9, 132.3, 132.4, 136.2, 136.7, 140.2, 141.4, 147.2, 152.9, 163.2. LC/MS (ESI): *m*/*z* = 601 (M^+^); anal. calcd for C_33_H_24_BrN_5_O_2_: C, 65.79; H, 4.02; N, 11.62; found: C, 65.86; H, 4.11; N, 11.73.

#### 4.1.3 3-((3′-(2-Chlorohenyl)-4′-nitrospiro[indeno[1,2-*b*]quinoxaline-11,2′-pyrrolidin]-5′-yl)methyl)-1*H*-indole, 6c

White solid; Mp: 228–240 °C; ^1^H NMR (DMSO-d_6_, 400 MHz): *δ*/ppm 2.92–2.98 (dd, *J* = 14.0, 9.6 Hz, 1H), 3.04–3.09 (dd, *J* = 14.0, 4.4 Hz, 1H), 5.26–5.31 (m, 1H), 5.51 (d, *J* = 8.0 Hz, 1H), 6.95 (t, *J* = 9.2 Hz, 1H), 6.48–6.51 (m, 1H, ArH), 6.61–6.65 (m, 1H, ArH), 6.81 (d, *J* = 8.4 Hz, 1H, ArH), 6.92–6.99 (m, 4H, ArH), 7.14–7.17 (m, 1H, ArH), 7.24–7.28 (m, 1H, ArH), 7.40–7.44 (m, 1H, ArH), 7.46–7.54 (m, 2H, ArH), 7.61–7.63 (m, 2H, ArH), 7.70–7.73 (m, 1H, ArH), 7.89–7.93 (m, 1H, ArH), 7.99 (d, *J* = 8.0 Hz, 1H), 9.47 (s, 1H, NH); ^13^C NMR (CDCl_3_, 100 MHz): *δ/*ppm 27.4, 53.4, 59.1, 72.0, 93.6, 110.1, 111.1, 118.2, 118.6, 121.2, 123.0, 125.5, 125.7, 126.8, 128.3, 128.5, 128.6, 128.8, 129.0, 129.2, 129.3, 129.5, 129.6, 131.1, 131.3, 134.4, 136.1, 136.4, 140.0, 141.5, 145.9, 152.8, 162.4. LC/MS(ESI): *m*/*z* = 557 (M^+^); anal. calcd for C_33_H_24_ClN_5_O_2_: C, 71.03; H, 4.34; N, 12.55; found: C, 71.12; H, 4.41; N, 12.63.

#### 4.1.4 3-((3′-(4-chlorohenyl)-4′-nitrospiro[indeno[1,2-*b*]quinoxaline-11,2′-pyrrolidin]-5′-yl)methyl)-1*H*-indole, 6d

White solid; Mp: 212–214 °C; ^1^H NMR (DMSO-d_6_, 400 MHz): *δ*/ppm 2.55–2.61 (m, 2H), 4.36 (d, *J* = 9.6 Hz, 1H), 4.72–4.82 (m, 1H), 6.06 (t, *J* = 9.2 Hz, 1H), 6.23 (d, *J* = 8.0 Hz, 2H, ArH), 6.32 (d, *J* = 8.4 Hz, 2H, ArH), 6.59–6.62 (m, 2H, ArH), 6.69–6.70 (m, 1H, ArH), 6.85 (d, *J* = 6.6 Hz, 1H, ArH), 7.02 (t, *J* = 7.2 Hz, 1H, ArH), 7.22–7.25 (m, 4H, ArH), 7.34 (d, *J* = 7.6 Hz, 1H, ArH), 7.41 (d, *J* = 6.4 Hz, 1H, ArH), 7.62 (d, *J* = 6.4 Hz, 2H, ArH), 9.87 (s, 1H, NH); ^13^C NMR (CDCl_3_, 100 MHz): *δ*/ppm 27.2, 56.5, 56.8, 70.9, 89.8, 109.1, 110.4, 117.4, 117.6, 120.2, 123.5, 126.2, 127.0, 127.9, 128.3, 128.6, 128.7, 129.1, 130.5, 131.2, 132.1, 135.4, 136.0, 139.5, 140.8, 145.7, 151.8, 157.4, 161.7. LC/MS (ESI): *m/z* = 557 (M^+^); anal. Calcd for C_33_H_24_ClN_5_O_2_: C, 71.03; H, 4.34; N, 12.55; found: C, 71.11; H, 4.43; N, 12.62.

#### 4.1.5 3-((3′-(4-Methylphenyl)-4′-nitrospiro[indeno[1,2-*b*]quinoxaline-11,2′-pyrrolidin]-5′-yl)methyl)-1*H*-indole, 6e

White solid; Mp: 186–184 °C; ^1^H NMR (DMSO-d_6_, 400 MHz): *δ*/ppm 1.91 (s, 3H), 3.02–3.11 (m, 2H), 4.03 (d, *J* = 6.8 Hz, 1H, NH), 4.82 (d, *J* = 10.4 Hz, 1H), 5.07–5.12 (m, 1H), 6.61–6.71 (m, 5H), 7.02–7.09 (m, 2H, ArH), 7.23 (m, 1H, ArH), 7.34 (d, *J* = 8.0 Hz, 1H, ArH), 7.54 (t, *J* = 7.2 Hz, 1H, ArH), 7.54 (t, *J* = 7.2 Hz, 1H, ArH), 7.68–7.78 (m, 4H, ArH), 7.84 (d, *J* = 7.2 Hz, 1H, ArH), 7.94–7.97 (m, 1H, ArH), 8.16–8.19 (m, 1H, ArH), 10.90 (s, 1H, NH); ^13^C NMR (DMSO-d_6_, 100 MHz): *δ/*ppm 20.2, 28.4, 57.1, 71.8, 90.7, 110.0, 118.3, 118.4, 120.9, 121.4, 123.6, 125.2, 127.3, 127.6, 128.6, 128.7, 129.2, 129.3, 129.7, 129.8, 132.2, 136.2, 136.7, 136.8, 140.3, 141.4, 147.6, 153.0, 163.6. LC/MS(ESI): *m*/*z* = 537 (M^+^); anal. calcd for C_34_H_27_N_5_O_2_: C, 75.96; H, 5.06; N, 13.03; found: C, 76.05; H, 5.18; N, 13.12.

#### 4.1.6 3-((3′-(2-Methoxyphenyl)-4′-nitrospiro[indeno[1,2-*b*]quinoxaline-11,2′-pyrrolidin]-5′-yl)methyl)-1*H*-indole, 6f

White solid; Mp: 199–201 °C; ^1^H NMR (DMSO-d_6_, 400 MHz): *δ*/ppm 3.03–3.13 (m, 2H), 3.21 (s, 3H), 4.03 (d, *J* = 5.6 Hz, 1H, NH), 5.21–5.23 (m, 1H), 5.45 (d, *J* = 8.8 Hz, 1H), 6.35 (t, *J* = 7.6 Hz, 1H), 6.54–6.60 (m, 2H, ArH), 6.83 (t, *J* = 8.0 Hz, 1H, ArH) 6.92 (d, *J* = 7.2 Hz, 1H, ArH), 7.02–7.08 (m, 2H, ArH), 7.25 (m, 1H, ArH), 7.33–7.35 (m, 1H, ArH), 7.51 (t, *J* = 7.2 Hz, 1H, ArH), 6.71–6.77 (m, 4H, ArH), 7.82 (d, *J* = 7.2 Hz, 1H, ArH), 7.94 (d, *J* = 7.2 Hz, 1H, ArH), 7.14–7.16 (m, 2H, ArH), 10.86 (s, 1H, NH); ^13^C NMR (DMSO-d_6_, 100 MHz): *δ/*ppm 28.1, 50.3, 54.8, 58.2, 71.8, 91.7, 110.1, 110.8, 111.4, 118.3, 119.5, 120.9, 121.3, 123.6, 126.0, 127.3, 128.1, 128.5, 128.6, 129.1, 129.2, 129.4, 129.6, 131.5, 136.3, 140.1, 141.3, 148.1, 153.1, 157.1, 164.0. LC/MS (ESI): *m/z* = 553 (M^+^); anal. calcd for C_34_H_27_N_5_O_3_: C, 73.76; H, 4.92; N, 12.65; found: C, 73.87; H, 4.85; N, 12.54.

#### 4.1.7 3-((3′-(3-Methoxyphenyl)-4′-nitrospiro[indeno[1,2-*b*]quinoxaline-11,2′-pyrrolidin]-5′-yl)methyl)-1*H*-indole, 6g

White solid; Mp: 276–278 °C; ^1^H NMR (DMSO-d_6_, 400 MHz): *δ*/ppm 3.08–3.15 (m, 2H), 3.23 (s, 3H), 4.07 (d, *J* = 6.0 Hz, 1H, NH), 4.86 (d, *J* = 10.4 Hz, 1H), 5.11–5.15 (m, 1H), 6.29 (m, 1H), 6.38 (d, *J* = 8.0 Hz, 1H, ArH), 6.46 (d, *J* = 8.0 Hz, 1H, ArH), 6.69–6.79 (m, 2H, ArH), 7.02–7.10 (m, 2H, ArH), 7.26 (m, 1H, ArH), 7.36 (d, *J* = 8.0 Hz, 1H, ArH), 7.55 (t, *J* = 7.2 Hz, 1H, ArH), 7.68–7.77 (m, 4H, ArH), 7.85 (d, *J* = 8.0 Hz, 1H, ArH), 7.97 (d, *J* = 7.6 Hz, 1H, ArH), 8.17–8.21 (m, 2H, ArH), 10.87 (s, 1H, NH); ^13^C NMR (DMSO-d_6_, 100 MHz): *δ/*ppm 28.4, 50.5, 54.2, 57.6, 71.9, 90.7, 110.0, 111.3, 111.5, 113.0, 113.4, 118.5, 120.9, 121.2, 121.4, 123.8, 123.9, 125.2, 127.3, 129.2, 129.6, 129.9, 132.5, 134.4, 136.3, 136.8, 140.3, 141.4, 147.6, 153.0, 158.5, 163.6. LC/MS (ESI): *m*/*z* = 553 (M^+^); anal. calcd for C_34_H_27_N_5_O_3_: C, 73.76; H, 4.92; N, 12.65; found: C, 73.84; H, 4.98; N, 12.71.

#### 4.1.8 3-((3′-(4-Methoxyphenyl)-4′-nitrospiro[indeno[1,2-*b*]quinoxaline-11,2′-pyrrolidin]-5′-yl)methyl)-1*H*-indole, 6h

White solid; Mp: 262–264 °C; ^1^H NMR (DMSO-d_6_, 400 MHz): *δ*/ppm 3.02–3.11 (m, 2H), 3.41 (s, 3H), 4.03 (d, *J* = 6.4 Hz, 1H, NH), 4.81 (d, *J* = 10.0 Hz, 1H), 5.05–5.10 (m, 1H), 6.39 (m, 2H, ArH), 6.49 (t, *J* = 9.6 Hz, 1H, ArH), 6.67–6.70 (m, 2H, ArH), 7.00–7.09 (m, 2H, ArH), 7.23–7.24 (m, 1H, ArH), 7.34 (d, *J* = 8.0 Hz, 1H, ArH), 7.54 (t, *J* = 8.0 Hz, 1H, ArH), 7.68–7.78 (m, 4H, ArH), 7.85 (d, *J* = 8.0 Hz, 1H, ArH), 7.97 (d, *J* = 7.6 Hz, 1H, ArH), 8.17–8.20 (m, 2H, ArH), 10.87 (s, 1H, NH); ^13^C NMR (DMSO-d_6_, 100 MHz): *δ/*ppm 28.4, 54.7, 56.8, 57.0, 71.8, 90.9, 110.0, 111.4, 113.4, 118.3, 120.9, 121.4, 123.6, 124.5, 125.2, 127.3, 128.7, 128.9, 129.2, 129.3, 129.7, 129.8, 132.2, 136.2, 136.7, 140.3, 141.4, 147.6, 153.1, 158.4, 163.7. LC/MS (ESI): *m/z* = 553 (M^+^); anal. calcd for C_34_H_27_N_5_O_3_: C, 73.76; H, 4.92; N, 12.65; found: C, 73.88; H, 4.84; N, 12.76.

#### 4.1.9 3-((3′-(2-Fluorophenyl)-4′-nitrospiro[indeno[1,2-*b*]quinoxaline-11,2′-pyrrolidin]-5′-yl)methyl)-1*H*-indole, 6i

White solid; Mp: 175–177 °C; ^1^H NMR (DMSO-d_6_, 400 MHz): *δ*/ppm 3.01–3.07 (dd, *J* = 13.6, 8.8 Hz, 1H), 3.11–3.20 (m, 1H), 4.11 (d, *J* = 6.4 Hz, 1H, NH), 5.18–5.22 (m, 1H), 5.28 (d, *J* = 9.6 Hz, 1H), 6.61–6.68 (m, 2H, ArH), 6.99 (t, *J* = 9.6 Hz, 1H, ArH), 6.91–6.94 (m, 1H, ArH), 7.01–7.12 (m, 3H, ArH), 7.26–7.27 (m, 1H, ArH), 7.34 (d, *J* = 8.0 Hz, 1H, ArH), 7.53 (t, *J* = 8.0 Hz, 1H, ArH), 7.71–7.78 (m, 3H, ArH), 7.83 (d, *J* = 8.0 Hz, 1H, ArH), 7.96 (d, *J* = 7.6 Hz, 1H, ArH), 8.13–8.19 (m, 3H, ArH); ^13^C NMR (DMSO-d_6_, 100 MHz): *δ/*ppm 28.2, 58.3, 63.9, 71.7, 91.2, 109.9, 110.7, 111.2, 118.3, 118.5, 120.1, 120.2, 121.0, 121.2, 123.8, 123.6, 125.5, 127.3, 129.3, 129.5, 129.7, 132.3, 136.3, 136.4, 136.6, 140.2, 141.5, 147.2, 153.0, 159.0, 160.3 (*J* = 246.2 Hz), 163.4. LC/MS (ESI): *m*/*z* = 553 (M^+^); anal. calcd for C_33_H_24_FN_5_O_2_: C, 73.19; H, 4.47; N, 12.93; found: C, 73.30; H, 4.60; N, 13.02.

#### 4.1.10 3-((3′-(4-Fluorophenyl)-4′-nitrospiro[indeno[1,2-*b*]quinoxaline-11,2′-pyrrolidin]-5′-yl)methyl)-1*H*-indole, 6j

White solid; Mp: 168–170 °C; ^1^H NMR (DMSO-d_6_, 400 MHz): *δ*/ppm 3.02–3.13 (m, 2H), 4.06 (d, *J* = 6.6 Hz, 1H, NH), 4.99 (d, *J* = 9.6 Hz, 1H), 5.09–5.13 (m, 1H), 6.67–6.72 (m, 3H, ArH), 6.81–6.84 (m, 2H, ArH), 7.00–7.09 (m, 2H, ArH), 7.24–7.25 (m, 1H, ArH), 7.35 (d, *J* = 7.2 Hz, 1H, ArH), 7.56 (t, *J* = 8.0 Hz, 1H, ArH), 7.69–7.77 (m, 4H, ArH), 7.86 (d, *J* = 8.0 Hz, 1H, ArH), 7.97 (d, *J* = 7.6 Hz, 1H, ArH), 8.16–8.22 (m, 2H, ArH); ^13^C NMR (DMSO-d_6_, 100 MHz): *δ*/ppm 28.3, 56.6, 57.3, 71.8, 90.6, 109.9, 111.4, 114.7, 114.9, 118.3, 118.4, 120.9, 121.5, 123.6, 125.3, 127.3, 128.7, 129.2, 129.7, 129.8, 129.9, 130.0, 132.3, 136.2, 136.7, 140.3, 141.4, 147.3, 153.0, 161.3 (*J* = 243.0 Hz), 163.4. LC/MS (ESI): *m*/*z* = 553 (M^+^); anal. calcd for C_33_H_24_FN_5_O_2_: C, 73.19; H, 4.47; N, 12.93; found: C, 73.28; H, 4.56; N, 13.01.

#### 4.1.11 3-((3′-(3-Nitrophenyl)-4′-nitrospiro[indeno[1,2-*b*]quinoxaline-11,2′-pyrrolidin]-5′-yl)methyl)-1*H*-indole, 6k

White solid; Mp: 254–256 °C; ^1^H NMR (DMSO-d_6_, 500 MHz): *δ*/ppm 3.00–3.05 (dd, *J* = 14.0, 9.0 Hz, 1H), 3.09–3.13 (dd, *J* = 14.5, 4.5 Hz, 1H), 4.10 (d, *J* = 6.0 Hz, 1H, NH), 5.04 (d, *J* = 10.0 Hz, 1H), 5.13–5.19 (m, 1H), 6.75 (t, *J* = 9.0 Hz, 1H), 6.99–7.06 (m, 2H, ArH), 7.11 (t, *J* = 8.0 Hz, 1H, ArH), 7.23–7.26 (m, 2H, ArH), 7.32 (d, *J* = 8.0 Hz, 1H, ArH), 7.54 (t, *J* = 7.0 Hz, 1H, ArH), 7.64–7.65 (m, 1H, ArH), 7.69–7.72 (m, 3H, ArH), 7.73–7.78 (m, 2H, ArH), 7.81 (d, *J* = 7.5 Hz, 1H, ArH), 7.88–7.90 (m, 1H, ArH), 8.15–8.17 (m, 1H, ArH), 8.24 (d, *J* = 7.5 Hz, 1H, ArH), 10.84 (s, 1H, NH); ^13^C NMR (DMSO-d_6_, 125 MHz): *δ/*ppm 28.6, 57.3, 58.5, 72.5, 91.0, 110.5, 111.9, 118.9, 119.0, 121.5, 122.0, 123.0, 123.1, 124.2, 125.9, 127.9, 129.2, 129.8, 129.9, 130.0, 130.5, 130.7, 133.0, 135.1, 136.0, 136.8, 137.2, 140.8, 141.9, 147.4, 147.5, 153.3, 163.5. LC/MS (ESI): *m/z* = 568 (M^+^); anal. calcd for C_33_H_24_N_6_O_4_: C, 69.71; H, 4.25; N, 14.78; found: C, 69.80; H, 4.33; N, 14.85.

## Conflicts of interest

There are no conflicts to declare.

## Supplementary Material

RA-010-D0RA02525A-s001

RA-010-D0RA02525A-s002
