# Supplementary material for: Regio- and diastereoselective synthesis of spiropyrroloquinoxaline grafted indole heterocyclic hybrids and evaluation of their anti-Mycobacterium tuberculosis activity
Source: RSC Adv. 2020 Jun 19;10(40):23522–31. doi: 10.1039/d0ra02525a (PMC9054781; doi:10.1039/d0ra02525a)
Supplement: RA-010-D0RA02525A-s001 [file RA-010-D0RA02525A-s001.pdf]

Electronic Supplementary Material (ESI) for RSC Advances.
This journal is © The Royal Society of Chemistry 2020

Regio- and diastereoselective synthesis of spiropyrroloquinoxaline grafted indole hetercyclic hybrids and evaluation of their anti-Mycobacterium tuberculosis activity

Natarajan Arumugam[a,*], Abdulrahman I. Almansour[a], Raju Suresh Kumar[a], Abdul Jaleel Mohammad Ali Al-Aizari Shatha Ibrahim Alaqeel[b], Sevgi Kansız[c], Vagolu Siva Krishna,[d] Dharmarajan Sriram,[d] Necmi Dege[e]

[a]*Department of Chemistry, College of Science, King Saud University, P.O Box 2455, Riyadh 11451, Saudi Arabia*

[b]*Department of Chemistry, College of Science, King Saud University (034), Riyadh 11495, Saudi Arabia.*

[c]*Department of Fundamental Sciences, Faculty of Engineering, Samsun University, Samsun, 55420, Turkey*

[d]*Medicinal Chemistry and Antimycobacterial Research Laboratory, Pharmacy Group, Birla Institute of Technology & Science-Pilani, Hyderabad Campus, Jawahar Nagar, Hyderabad 500078, Telangana, India.*

[e]*Department of Physics, Faculty of Arts and Sciences, Ondokuz Mayıs University, Samsun, 55139, Turkey*

S. No	List of Figures	Page No
**1**	^{1}H NMR spectrum of **6k**	**6**
**2**	Expanded ^{1}H NMR spectrum of **6k**	**6**
**3**	^{13}C NMR spectrum of **6k**	**7**
**4**	DEPT-135 spectrum of **6k**	**8**
**5**	^{1}H,^{1}H-COSY spectrum of **6k**	**9**
**6**	Expanded ^{1}H,^{1}H-COSY spectrum of **6k**	**10**
**7**	^{13}C, ^{1}H-COSY(HMQC) spectrum of **6k**	**11**
**8**	Expanded ^{13}C, ^{1}H-COSY(HMQC) spectrum of **6k**	**12**
**9**	HMBC spectrum of **6k**	**13**
**10**	Expanded HMBC spectrum of **6k**	**14**

Table 1. Crystal data and structure refinement parameters for **6e.**

**Crystal Data**	
Chemical Formula	$C_{34}H_{27}N_5O_2$
Formula weight (a.k.b.)	537.60
Temperature (K)	296
Crystal system	Triclinic
Space group	*P-1*
Unit cell parameters	
a, b, c (Å)	11.5002 (7), 11.7543 (8), 14.3642 (10)
α , β, γ (˚)	97.266 (5), 106.616 (5), 111.900 (5)
Crystal size (mm)	0.39 × 0.29 × 0.23
Volume, V ($Å^3$)	1665.9 (2)
Z	2
μ (mm^{-1})	0.07
F_{000}	564
Calculated density (Mg/m^3)	1.072
**Data collection**	
Diffractometer	STOE IPDS 2
Wavelength (Å)	0.71073
θ range for data collection (˚)	$2.1 \leq \theta \leq 26.0$
Index ranges	
h_{min}, h_{max}; k_{min}, k_{max}; l_{min}, l_{max}	-13, 14; -14, 14; -17, 17
Measurement method	ω scan
Reflections collected	16765
Independent reflections	6550
Observed reflections [I > 2σ(I)]	3270
Absorption correction	Integration
T_{min}, T_{max}	0.9732, 0.9866
R_{int}	0.051
**Refinement**	
Refinement method	SHELXL17/1
Parameters	375
$R[F^2 > 2\sigma(F^2)]$	0.057
$wR(F^2)$	0.152
GooF = S	0.94
$\Delta\rho_{min}$, $\Delta\rho_{max}$ ($e/Å^3$)	-0.10, 0.17

Table 2. Hydrogen bonding geometry for **6e**.

D─H···A	*D*─H	H···A	*D*···A	*D*─H···A
C18─H18···N2	0.93	2.61	3.338 (3)	135
C24─H24···O2^{i}	0.98	2.56	3.433 (3)	148
N3─H3···N1^{ii}	0.99(2)	2.35(3)	3.260 (3)	153.5 (2)

Symmetry codes: (*i*) –*x*+*2*, −*y*, −*z*+1; (*ii*) −*x*+1, −*y*−1, −*z*+1.

General Method

Chemistry

^{1}H and ^{13}C NMR spectra were recorded on a Varian Mercury JEOL-400/500 NMR spectrometers in DMSO-d_6 using TMS as internal standard. Chemical shifts are given in parts per million (δ-scale) and coupling constants are given in hertz. Elemental analyses were performed on a Perkin Elmer 2400 Series II Elemental CHNS analyzer.

Biology

In vitro anti tubercular activity

Briefly, the *Mycobacterium tuberculosis* H37Rv inoculum was prepared from fresh LJ medium re-suspended in 7H9-S medium (7H9 broth, 0.1% casitone, 0.5% glycerol, supplemented oleic acid, albumin, dextrose, and catalase [OADC]), adjusted to a OD_{590} 1.0, and diluted 1:20; 100 µl was used as inoculum. Each drug stock solution was thawed and diluted in 7H9-S at four-fold the final highest concentration tested. Serial two-fold dilutions of each drug were prepared directly in a sterile 96-well microtiter plate using 100 µl 7H9-S. A growth control containing no antibiotic and a sterile control were also prepared on each plate. Sterile water was added to all perimetre wells to avoid evaporation during the incubation. The plate was covered, sealed in plastic bags and incubated at 37°C in normal atmosphere. After 7 days incubation, 30 µl of alamar blue solution was added to each well, and the plate was re-incubated overnight. A change in colour from blue (oxidised state) to pink (reduced) indicated the growth of bacteria, and the MIC was defined as the lowest concentration of drug that prevented this change in colour [1,2].

In vitro cytotoxicity screening:

The *in vitro* cytotoxicity of the privileged antitubercular active analogues with lower MIC value were assessed by 3-(4,5- dimethylthiazol-2- yl)-2,5- diphenyltetrazolium bromide (MTT) assay against growth inhibition of RAW 264.7 cells at 50 µg/mL concentration.[3] Cell lines were maintained at 37°C in a humidified 5% CO_2 incubator (Thermo scientific). Detached the adhered cells and followed by centrifugation to get cell pellet. Fresh media was added to the pellet to make a cell count using haemocytometer and plate 100µl of

media with cells ranging from 5,000 - 6,000 per well in a 96-well plate. The plate was incubated overnight in CO_2 incubator for the cells to adhere and regain its shape. After 24hr cells were treated with the test compounds at 50 μg/mL diluted using the media to deduce the percentage inhibition on normal cells. The cells were incubated for 48 hr to assay the effect of the test compounds on different cell lines. Zero hour reading was noted down with untreated cells and also control with 1% DMSO to subtract further from the 48hr reading. After 48 hr incubation, cells were treated by MTT (4, 5-dimethylthiazol- 2-yl)- 2, 5-diphenyltetrazolium bromide) dissolved in PBS (5mg/ml) and incubated for 3-4 hr at 37°C. The formazan crystals thus formed were dissolved in 100μl of DMSO and the viability was measured at 540nm on a multimode reader (Spectra max). The values were further calculated for percentage inhibition which in turn helps us to know the cytotoxicity of the test compounds.

1. L.A. Collins, S.G. Franzblau, Antimicrob. Agents Chemother. 41 (1997), 1004-1009.
2. V.S. Krishna, S. Zheng, E. M. Rekha, L. W. Guddat, D. Sriram, Discovery and evaluation of novel Mycobacterium tuberculosis ketol-acid reductoisomerase inhibitors as therapeutic drug leads. J. Comput. Aided Mol. Des., 33 (2019) 357.
3. J. Van Meerloo, G.J. Kaspers, J. Cloos, Cell sensitivity assays: the MTT assay. Methods Mol. Biol 731 (2011) 237-245.

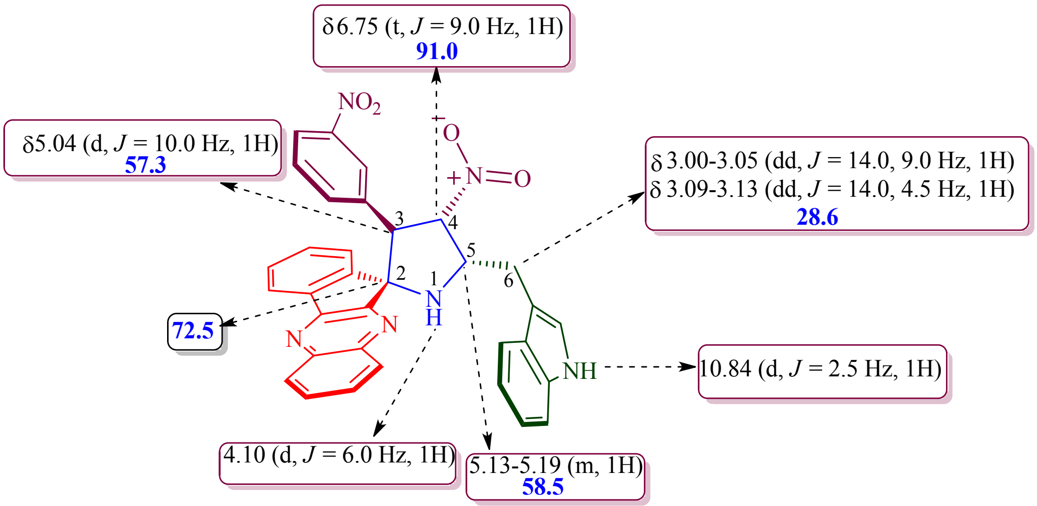

Figure S1. Selected ^{1}H and ^{13}C NMR Chemical shifts of **6k**

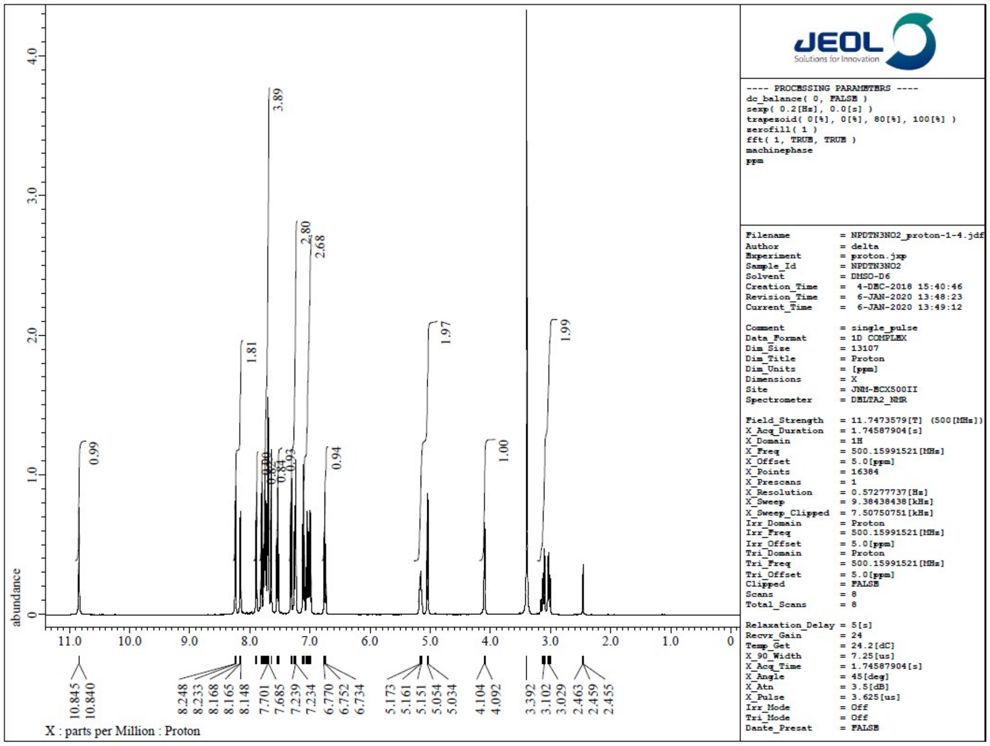

Figure S2. ^{1}H NMR spectrum of **6k**

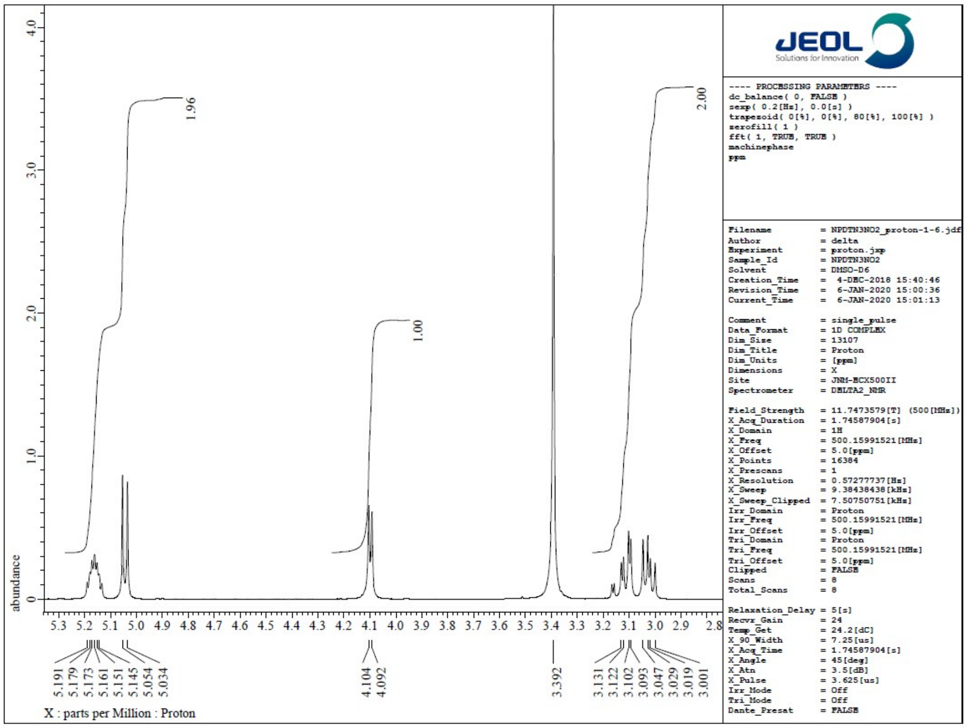

Figure S3. Expanded ^{1}H NMR spectrum of **6k**

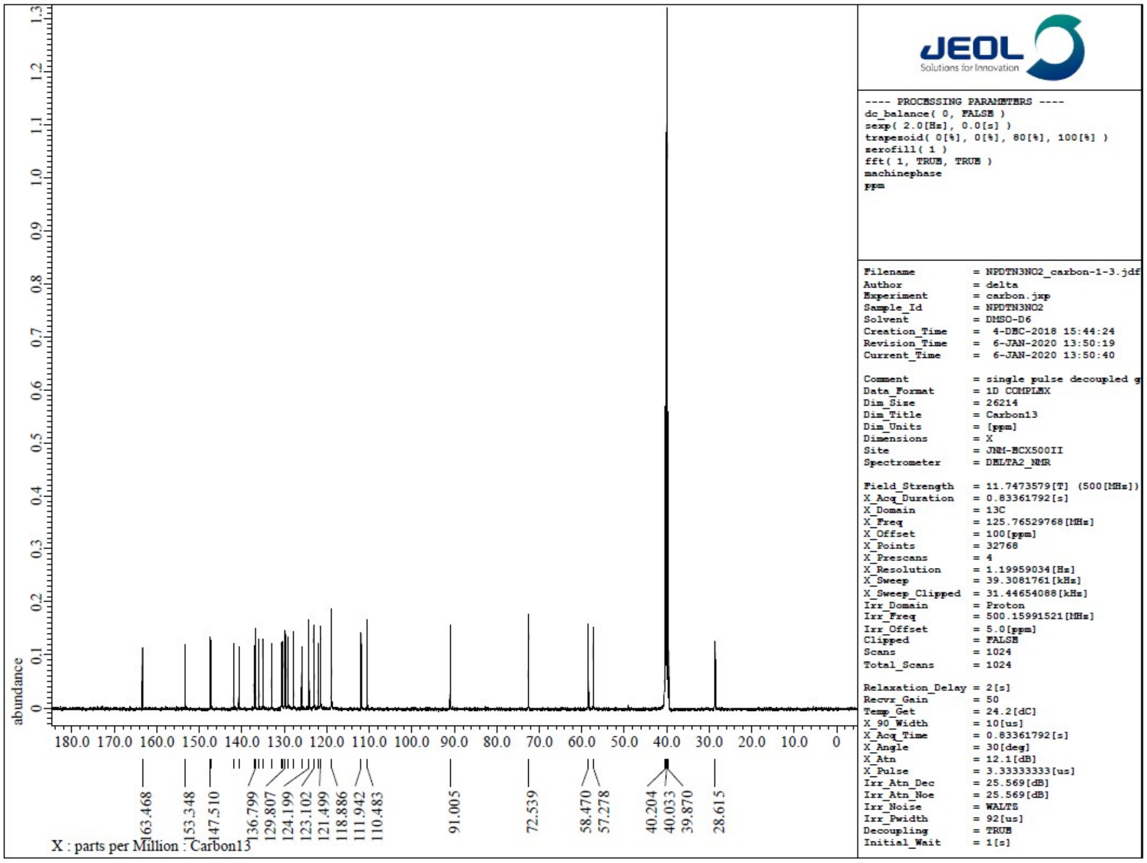

Figure S4. ^{13}C NMR spectrum of **6k**

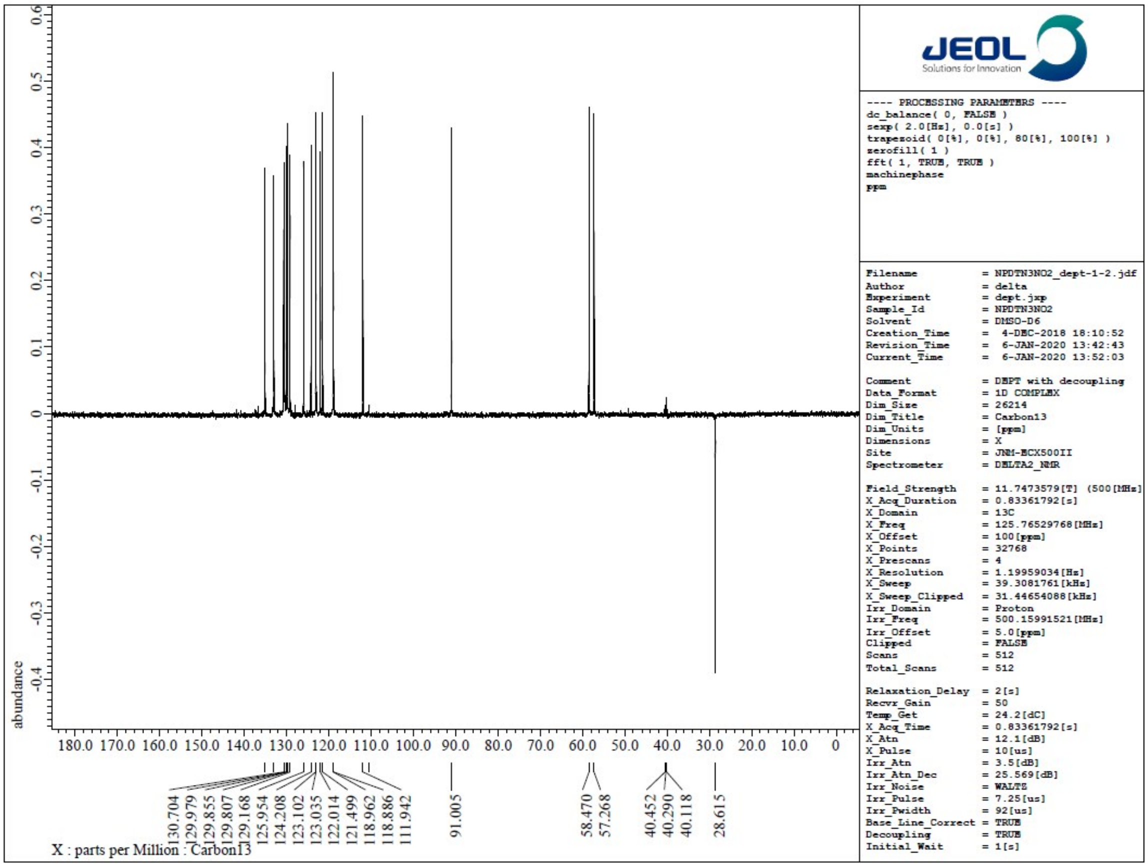

Figure S5. DEPT-135 spectrum of **6k**

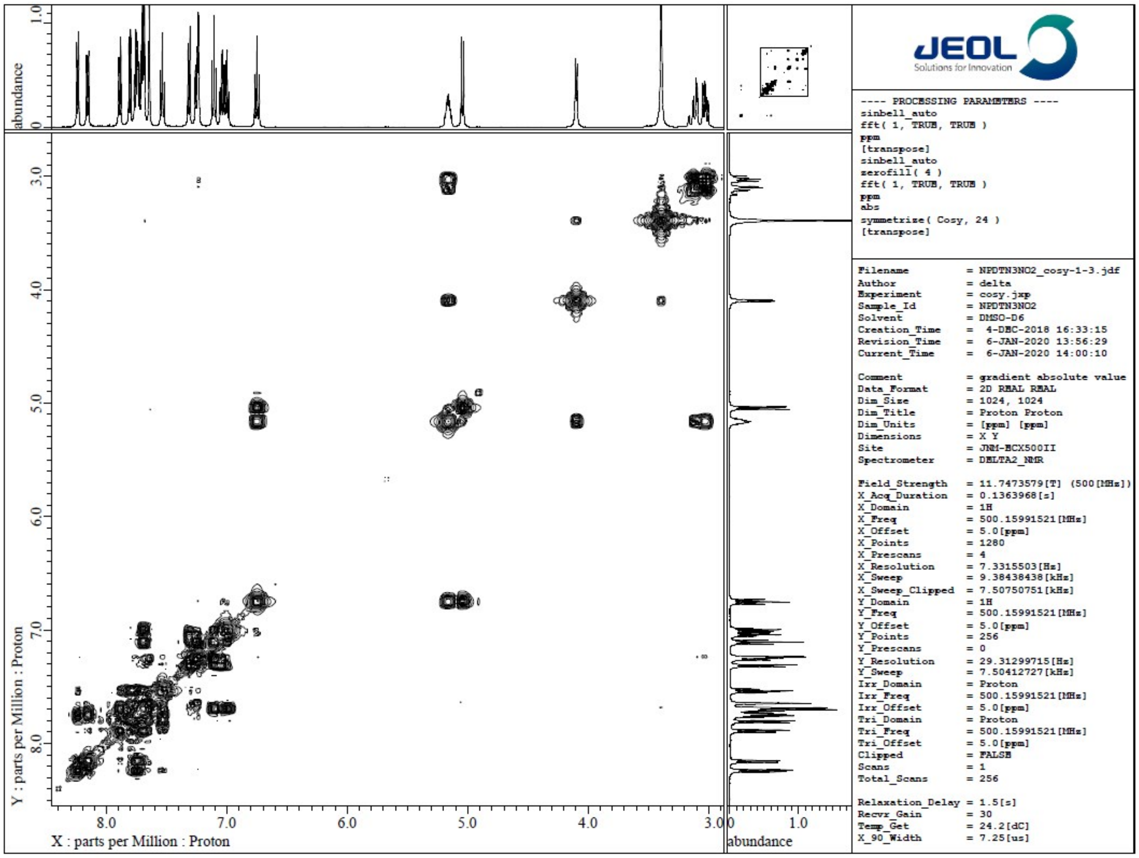

Figure S6. $^{1}H,^{1}H$-COSY spectrum of **6k**

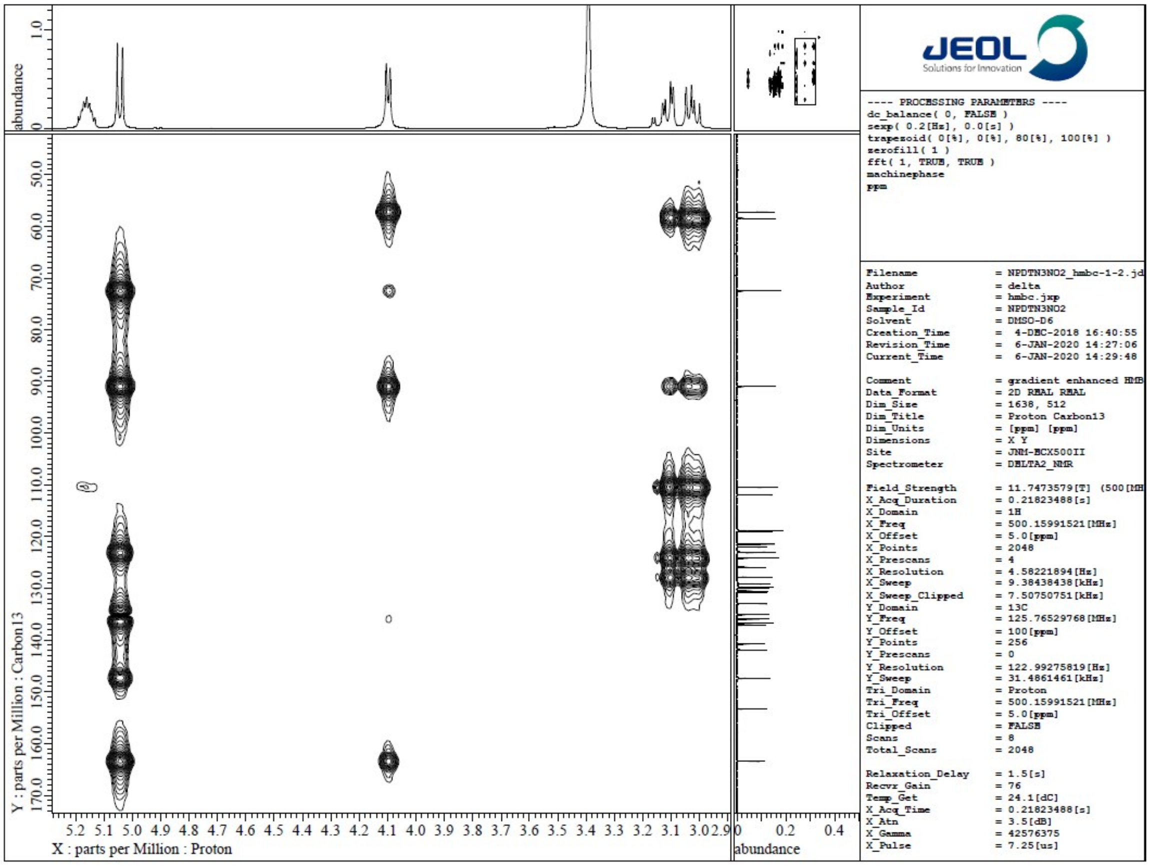

Figure S7. Expanded $^{1}H,^{1}H$-COSY spectrum of **6k**

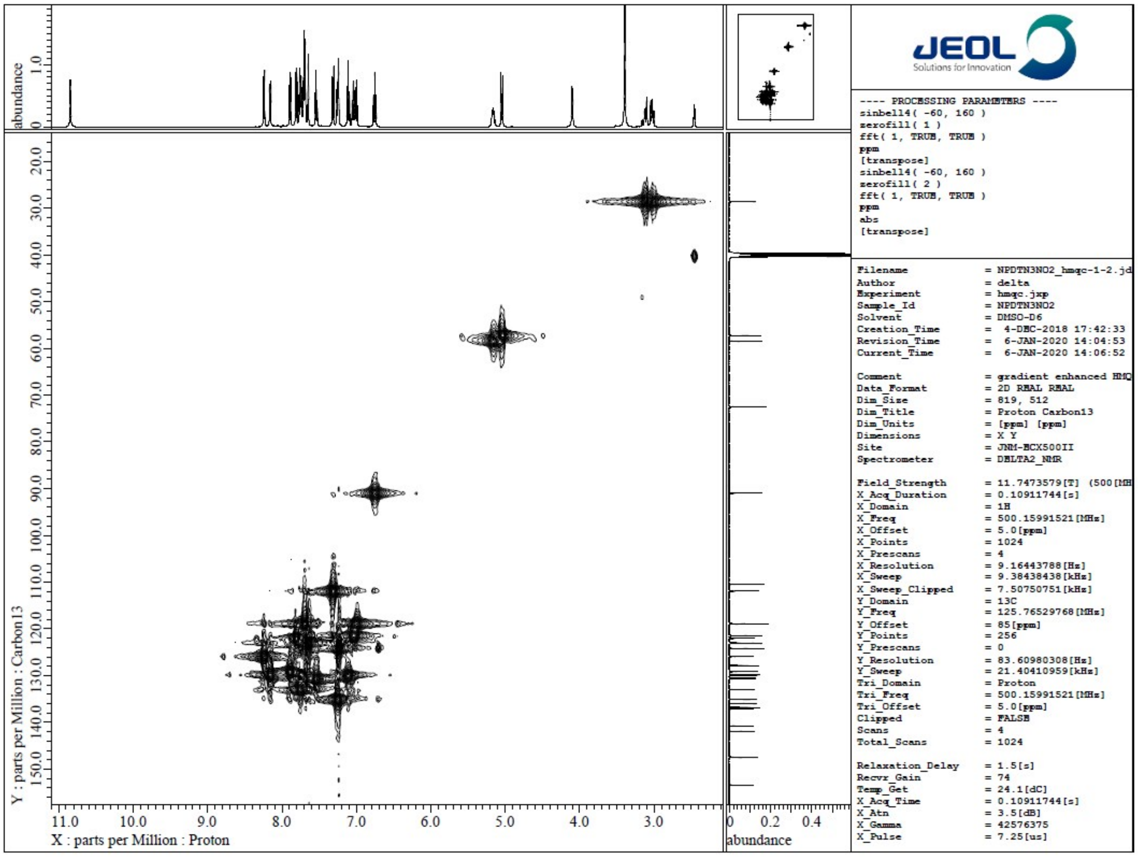

Figure S8. ^{13}C, ^{1}H,-COSY spectrum of **6k**

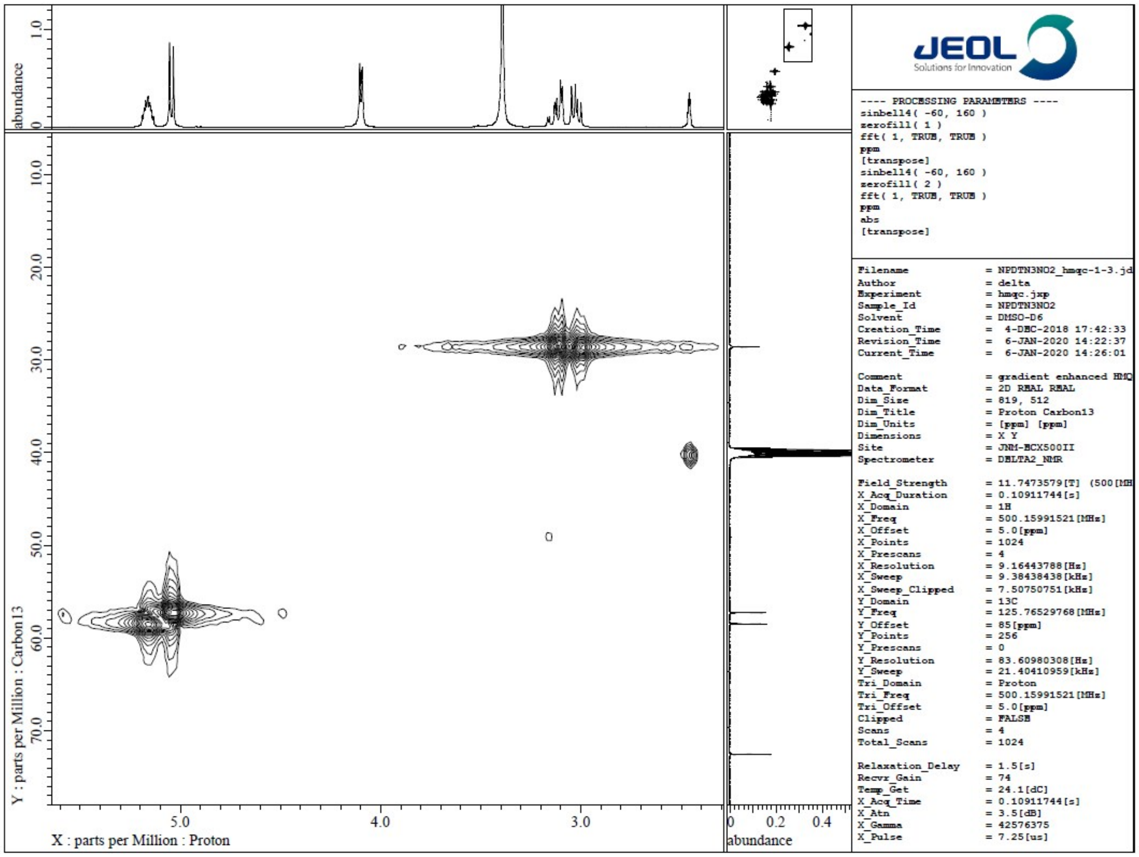

Figure S9. Expanded ^{13}C, ^{1}H-COSY spectrum of **6k**

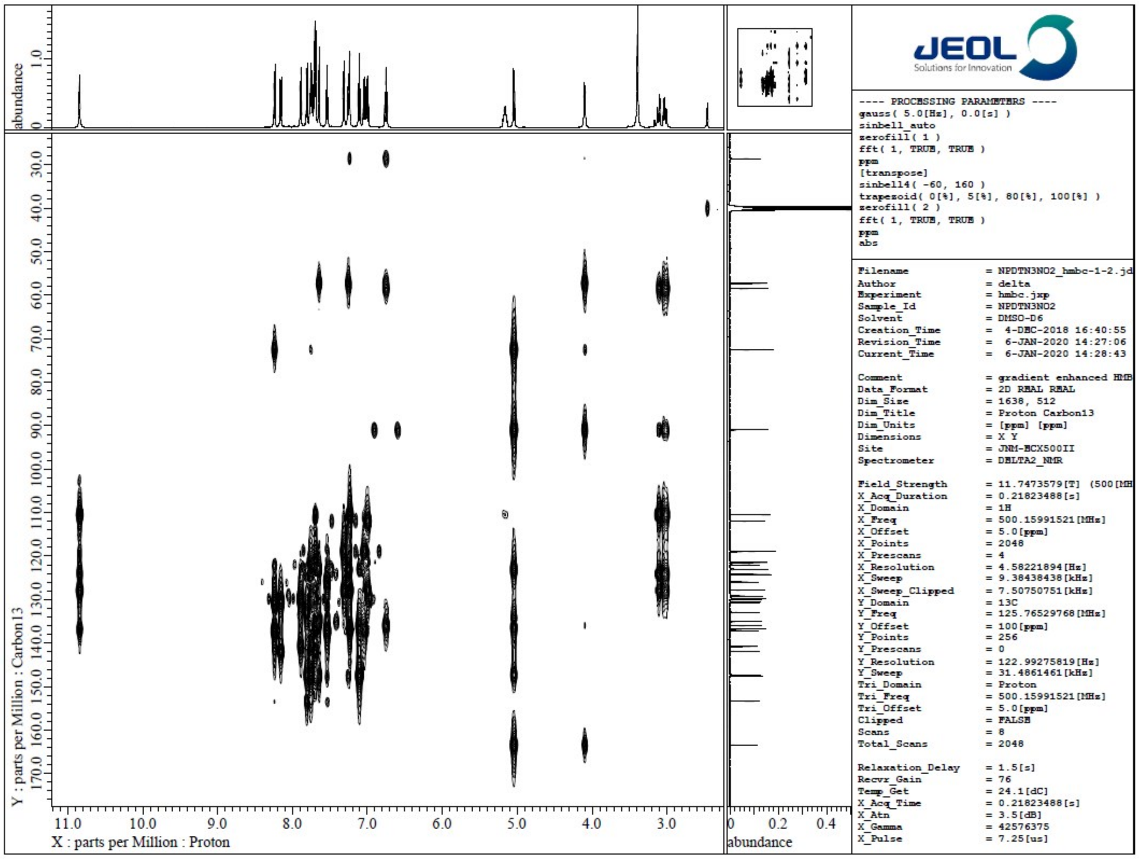

Figure S10. HMBC spectrum of **6k**

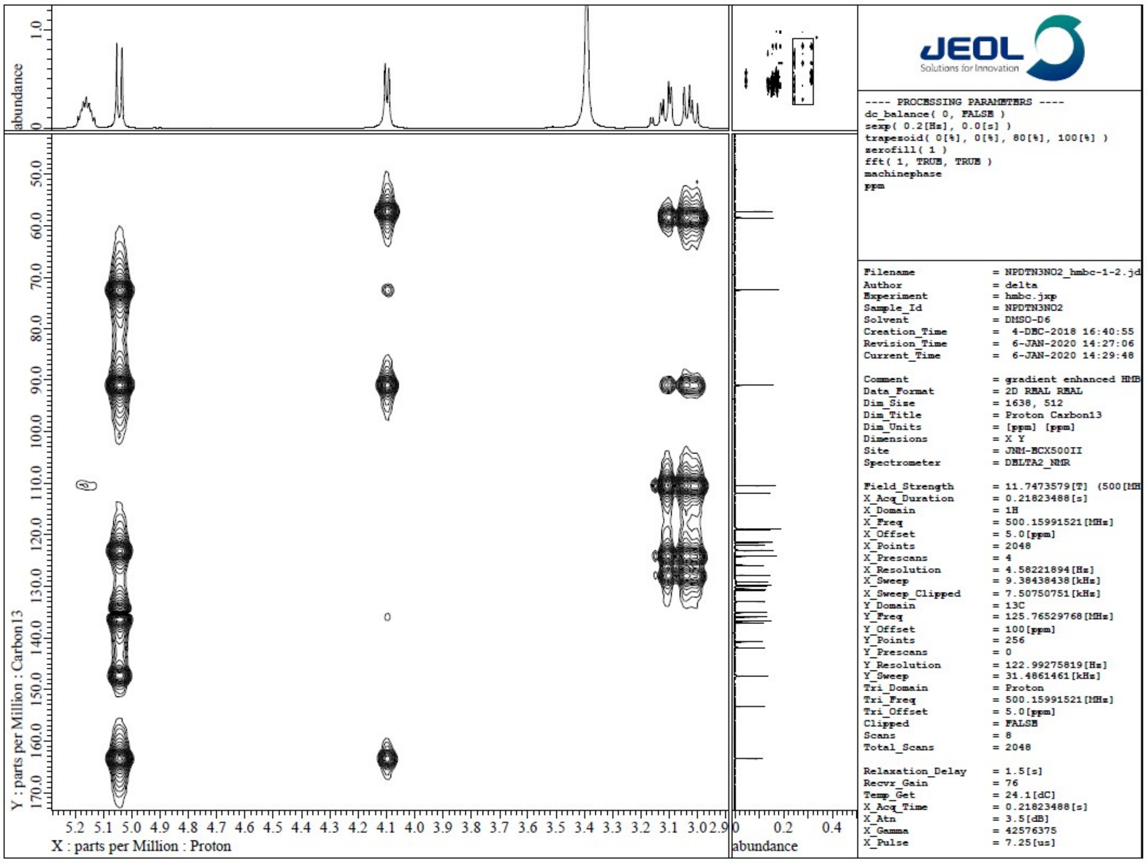

Figure S11. Expanded HMBC spectrum of **6k**
